# Time Production Intensively Studied in One Observer

**DOI:** 10.17505/jpor.2022.24219

**Published:** 2022-06-09

**Authors:** Joseph Glicksohn, Batsheva Weisinger

**Affiliations:** 1 Department of Criminology, Bar-Ilan University, Israel; 2 The Leslie and Susan Gonda (Goldschmied) Multidisciplinary Brain Research Center, Bar-Ilan University, Israel; 3 Stern College for Women, New York, USA

**Keywords:** time production, internal clock, psychophysical function, flicker, time perception

## Abstract

If one accepts the notion of an internal clock, then one must further presume that time production (TP) is attuned with the rate of functioning of the clock’s pacemaker. As the level of environmental stimulation increases, TP of the same target durations should decrease; this is particularly the case when one is exposed to flicker. In the present exploratory study, wherein the second author served in an *n* = 1 experiment, we intensely study TP, using a factorial design that crosses a factor of Flicker with one of Counting Strategy, to create 48 different conditions (sessions). In each session, 6 target intervals are produced a total of 6 times in a counterbalanced manner. Our results indicate that as flicker rate increases, produced duration decreases, as predicted. The main effect for flicker was found for the intercept, but not for the slope of the psychophysical function relating produced duration to target duration. Veridical perception is achieved at a flicker rate of 6 Hz. We uncovered no main effect for counting, suggesting that flicker swamps any impact of chronometric counting.

## Introduction

It is almost sixty years since the publication of Treisman’s (1963) influential model of an internal clock for time perception. In this model, an arousal-dependent pacemaker produces a sequence of pulses at a constant rate, which are subsequently stored, counted and ultimately transformed into an estimate of a time interval. Allan (1979, p. 349) noted some time ago, however, that although the Treisman paper is frequently cited, “there are no models which derive from it and little if any data have been analyzed in terms of it.” To our mind, this situation has changed radically during the past twenty-five years, on three counts.

First, the influential ‘attentional-gate’ model (Zakay & Block, 1997) is clearly an offshoot of Treisman’s model, as will be seen on comparing the two. The counter now is termed a ‘cognitive counter’, and short-term memory is now viewed as working memory, but essentially the cognitive architecture is preserved – though with the notable addition of both an attentional gate and a switch.

Second, Treisman’s model is undergoing refinement (Treisman et al., 1994), and he now suggests that the pacemaker consists of two components, each of which contributes separately to determining the pacemaker final output rate: The temporal oscillator (TO) and the calibration unit (CU) (Treisman & Brogan, 1992, p. 46).

Third, this renewed interest in an internal clock model is so because of the prominence of Scalar Expectancy Theory (SET), which is a particular instantiation of an internal clock model. SET is clearly a dominant paradigm in current research on time perception (Grondin, 2001; Matthews & Meck, 2016; Wearden, 1991; Wearden & Culpin, 1998). Wearden (2016, pp. 29-34) has recently presented the basic postulates of SET. These are as follows (using our own terminology): (1) An individual’s estimate (*P*) of a target duration (*T*) is unbiased and has an expected value of *T*; (2) the standard deviation (*SD*) of *P* is a linear function of *P*, hence is a linear function of *T*; (3) consequentially, the coefficient of variation (CV) is constant across *T*. Note that if *SD*(*P*) is a linear function of *P*, then *P* will not have a normal distribution. A logarithmic transformation of *P* will, however, normalize the distribution.

Consider, then, the following task: For eight seconds after seeing the word NOW, and without looking at your watch, stop reading and then tap the table! NOW. In this time production (TP) task, you produced the target duration (*P*) by signaling when that duration (*T*) is thought to have elapsed. For the required duration of 8 sec, individual **A** might produce a duration of 8 sec, individual **B** one of 10 sec, and individual **C** one of 6 sec. Note that for all three individuals, the produced duration (*P*) is subjectively viewed as lasting 8 sec (*T*). Individual **A** exhibits veridical time perception (i.e., *P* = *T*; 1 subjective second = 1 sec). Individual **B** would be viewed as having a slower internal clock (*P > T*), and individual **C** would be viewed as having a faster internal clock (*P < T*). Note, further, that instrumentation here can be minimal (a stopwatch) or elaborate (a computerized system) for running the task. Furthermore, the target duration or durations are not restricted to particular values; and the target interval can be ‘empty’ (e.g., Hancock & Block, 2016) or ‘filled’ (e.g., Baldauf et al., 2009) by whatever the experimenter chooses (continuous tone, intermittent flicker, a blue circle appearing on a screen, a movie clip, etc.). Note, further, that the participant can be instructed to count (e.g., Coelho et al., 2004), or not to count (e.g., Hicks & Allen, 1979), to close the eyes, or not to close the eyes, and so forth, depending on other considerations of the study.

In line with Treisman’s (1963) ‘internal clock’ model, the target duration (*T*; here 8 sec) in the TP task above corresponds to a specific number of stored pulses, or some function thereof (Treisman proposes a logarithmic function), which is subsequently used to decide when to delimit the un-folding of this duration – resulting in *P*. As Zakay (1993, p. 93) writes, *P* is based on a “predetermined number of subjective time units which are associated in one’s mind with the required objective time.” Or as Gibbon et al. (1997, p. 171) write, this decision is based on “the ratio of a currently evolving interval to a remembered standard.” Thus, a faster rate of functioning of the internal clock will lead to shorter productions, while a slower rate of functioning will lead to longer productions (Glicksohn, 2001). In the present exploratory study, we shall investigate whether an experimentally-induced faster rate of functioning of the internal clock does lead to shorter time productions.

The power function relating *P* to *T* (Eisler, 1976) is given by *P* = *aT*^β^, linearized as log(*P*) = log(*a*) + βlog(*T*) = α + βlog(*T*), α being the intercept, and β being the slope, and we have explicated the value of looking at TP data in terms of such a model elsewhere (Glicksohn & Hadad, 2012). Extending our example above to *T* values of 4, 8, 16, and 32 seconds, we note that if individual **A** exhibits respective *P* values of 4, 8, 16, and 32 seconds, the psychophysical function here would be characterized by α = 0, and β = 1. If individual **B** is consistent, exhibiting *P* values of 6, 10, 18, and 34 seconds, then the psychophysical function would have α ≠ 0, indicating a consistent bias (+2) in producing durations. When β ≠ 1, then the untransformed data are not consistent with a linear function. A TP task entailing a judicious choice of target durations will enable the investigation of the psychophysical function for time perception, which is preferable to a focus on a single duration (Eisler, 1996, p. 67). In the present study, we will be able to see to what degree performance on a TP task employing 6 target durations will be aptly fitted by such a psychophysical function. The second author served in an *n* = 1 study, having 48 sessions. Six target durations of 1, 2, 4, 8, 16 and 32 seconds served for the TP task, estimated using a completely balanced 6 x 6 Latin Square in each session. We shall present a detailed analysis of her data to see to what degree our TP task exhibits stability both within session and across sessions.

One factor underlying the structure of the 48 sessions is flicker rate. For if one accepts the notion of an internal clock (McAuley & Jones, 2003; Penney et al., 2000; van Rijn & Taatgen, 2008) then one must further presume that TP is attuned with the rate of functioning of the clock’s pacemaker (Baudouin et al., 2006; Boltz, 1994). One way to change the rate of functioning of the pacemaker is via the influence of exposure to environmental stimulation (Allman et al., 2014, p. 749). As the level of environmental stimulation increases, so TP of the same target durations will decrease (Glicksohn, 1992, 1996). This is particularly the case given exposure to visual or auditory flicker (Droit-Volet, 2014; Penton-Voak et al., 1996; Treisman & Brogan, 1992). We employ flicker in both the auditory and visual modalities. A modality effect could be indicative of a modality-specific pacemaker rate. For example, our data could very well show ‘auditory dominance’ whereby auditory flicker speeds up the pacemaker to a greater degree than does visual flicker (Chen & Yeh, 2009; Meck & Benson, 2002; Wearden et al., 1998). Alternatively, such auditory dominance might be viewed as indicating that there are separate clocks for each modality (Buhusi & Meck, 2009), and that the auditory pacemaker runs faster than does the visual pacemaker (Yuasa & Yotsumoto, 2015).

The benefits of employing flicker to study the rate of functioning of the pacemaker are threefold. First, as opposed to our previous studies investigating the influence of *prior* exposure to an altered sensory environment on *subsequent* TP (Glicksohn, 1992, 1996), we can investigate TP *while* exposed to flicker. Second, flicker stimulation enables the systematic variation of external tempo – namely, “the overall rate and frequency” (Boltz, 1994) of external stimulation – hence allowing for a parametric evaluation of the effects of flicker on TP. Third, our previous findings have highlighted the intercept (or, measure constant) of the psychophysical function relating TP to target duration, as being the locus of the effect of environmental stimulation (Glicksohn, 1996): As the level of environmental stimulation increases, so the intercept decreases. We can now investigate whether this finding regarding the intercept is replicable using flicker. Furthermore, we can see whether, as Rule (1993, p. 444) has argued, mean log(*P*) is a statistically more powerful measure than the intercept for evaluating experimental effects, or whether, as Wearden and Culpin (1998, p. 37) have suggested (albeit, using a linear and not a power function), the influence of flicker on the pacemaker should be found in the slope measure. Note that the linear function adopted by Wearden and colleagues comprises two additive components (Wearden et al., 1998, p. 104): the first is the slope measure, which will be affected by pacemaker rate, and the second is an intercept measure, which incorporates the difference in latency between opening and closing the switch of the accumulator. This linear function is used to measure the total number of pulses that have accumulated for a given *T* (Wearden, 2016, p. 60). The power function adopted in the present paper, linearized following logarithmic transformation, comprises two additive components: the first is the slope measure, which expresses the exponent of the power function relating *P* to *T*, and which will be close to 1 (when this exponent is exactly 1, then a linear function can be presumed), and the second is an intercept measure, which has been previously shown to indicate both trait and state/context effects on *P* (Glicksohn, 1996).

The second factor underlying the structure of the 48 sessions is chronometric counting. Chronometric counting (namely, “subdividing a given duration into a series of smaller intervals that are counted”; Allman et al., 2014, p. 754) improves performance (Hinton et al., 2004; Hinton & Rao, 2004; Ryan et al., 2004). This can be either by reducing intraindividual variance for *P* (Wearden, 1991), or by making this variance independent of *T* (Grondin, Ouellet, & Roussel, 2004). Why is the influence of chronometric counting on TP an important question to answer? This boils down to the very essence of how *P* is generated. Some researchers argue that chronometric counting should be discouraged (Kladopoulos et al., 2004; Mimura et al., 2000); others argue that this should be encouraged (Miró et al., 2003; Myers & Tilley, 2003). Note that while a longer *P* could be indicative of the fact that the internal clock is “…producing pulses at a considerably decreased rate” (Binkofski & Block, 1996, p. 491), it is more likely that “when humans are required to produce an integer number of seconds they count up to that integer value…” (Wearden, 1991, p. 71). Hence, in the present study we will be able to see to what degree performance on a TP task employing chronometric counting differs from performance when explicitly instructed not to use chronometric counting. In one condition, the TP tasks were performed while not counting. In three other conditions, chronometric counting was employed in 3 ways: Counting up to the target duration (‘one, two, three…’), counting down to the target duration (‘sixteen, fifteen, fourteen…’), and counting ‘one’ (‘one, one, one …’).

In the present study, therefore, we intensely study TP, using a factorial design that crosses a factor of Flicker with one of Counting Strategy, to create 48 different conditions (sessions). In each session, 6 target intervals are produced a total of 6 times in a counterbalanced manner. We stress that this is basically an exploratory study. The experimental protocol we present here, and the preliminary results reported here, can inform future studies employing time production that are concerned with the pacemaker rate of functioning.

## Method

### Participant and Design

The second author^1^ served in an *n* = 1 study, having 48 sessions, conforming to a Flicker (0 Hz, 6 Hz, 10 Hz, 18 Hz), Modality (Visual, Auditory, Visual & Auditory), and Counting Strategy (No counting, counting up, counting down, counting ‘one’) 4 × 3 × 4 fully crossed factorial design. The different sessions were completely randomized in their order of completion. To facilitate this, an instruction booklet was generated, with these 48 sessions each appearing on a separate page. Date and time of session were marked on each page. Up to seven successive sessions (with short breaks) were completed on any particular day. The study was completed within the space of a month.

### Flicker Stimulation

**Figure 1:**
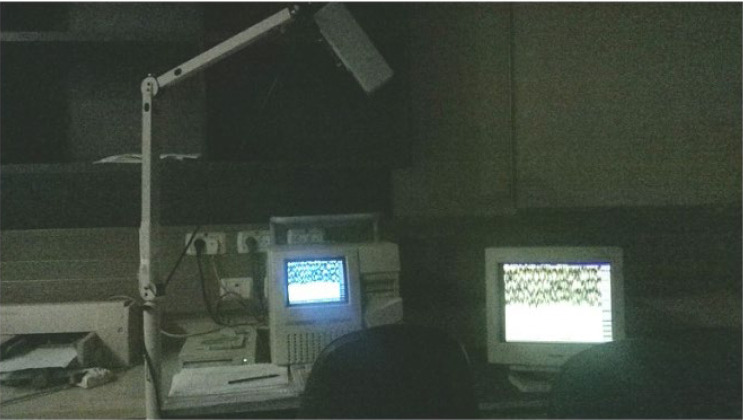
The experimental setup

Flicker stimulation was produced by the photo-stimulator of a Medelec DG Discovery digital EEG system. The lamp was positioned immediately above the participant (see Figure 1), who completed the task with eyes closed^2^ and when facing the screen and keyboard in front of her. Flicker at 6 Hz, 10 Hz and 18 Hz was produced by setting the flicker frequency to that particular rate of stimulation, as employed by the first author previously with this system (Glicksohn & Naftuliev, 2005). Flicker at 0 Hz comprised the ‘non-flicker’ control condition. In this condition, no light and no sound were produced by the lamp. The photo-stimulator provides simultaneous visual and auditory stimulation, namely photic stimulation (visual flicker) coupled with the sound of these clicks. To create unimodal visual flicker, the second author completed the relevant sessions with earphones on that muffled the sound of the clicks. To create unimodal auditory flicker, we covered the photo-stimulator with black cloth.

### Time Production

Six target durations of 1, 2, 4, 8, 16 and 32 seconds served for the time-production (TP) task. These were produced, with eyes closed, using a completely balanced 6 x 6 Latin Square in each session. Hence, each target duration was produced 6 times within a session; and each such duration was presented at a different ordinal position within a series of 6 target durations in that session. The second author was required to remain with her eyes closed while producing each of these target durations, by pressing and then releasing the ‘enter’ button on a keyboard. These data were recorded on line, with no immediate feedback as to how accurate she was on each trial. For each session, we employed a different ordering of the rows of the Latin Square.

### Counting strategies

In one condition, the second author completed the TP tasks while not counting. In three other conditions, she employed chronometric counting in one of three ways: Counting up to the target duration (‘one, two, three…’), counting down to the target duration (‘sixteen, fifteen, fourteen…’), and counting ‘one’ (‘one, one, one …’). Counting, it should be stressed, entailed explicit internal vocalization.

### Measures

Produced (*P*) and target (*T*) durations were both log-transformed (to base 2), rendering thereby a linear scale for both, ranging for *T* between 0 and 5, with a midpoint value of 2.5. Log(*P*) was then regressed on log(*T*), based on the 6 x 6 data points for that session, providing for each session an intercept value and a slope value, where the slope is equivalent to the exponent of the power function relating *P* to *T*. In addition, for each session we computed mean log(*P*), and we further computed within-session *SD* for each target duration, following log transformation of the data.

## Results

### General

Of the 48 data sets, 25 comprised complete data for analysis. Of the remaining 23 data sets, a total of 14 had missing data for the target duration of one second, due to a technical difficulty of the system in recording the data together with the ticks indicating the flicker rate for that duration. For the remaining 9 data sets, 4 had missing data (a target interval was inadvertently skipped during the session), there was 1 obvious blunder in reporting the produced duration (which was subsequently corrected), and there were 10 clear outliers that were subsequently discarded from the analysis.

The impact of this loss of data on the regression analyses is negligible, given that Log(*P*) was regressed on log(*T*) based on either the 6 x 6 data points comprising a complete data set for that session, or on the total number of usable data points for that session. Hence, there are 48 values for each of mean log(*P*), the slope and the intercept. Within-session *SD* for each target duration is, however, compromised by this loss of data. Of the 48 x 6 (target duration) *SD* values, 17 are missing for the target duration of one second, and 12 are missing for the other target durations. Rather than compute an *SD* value based on the data in hand (which would make their comparison rather problematic, each value being based on a different *n*), we preferred to ignore *SD* for the target duration of one second, and to allow for 12 missing values out of 240 for the remaining target durations.

Inspection of the individual psychophysical functions for each session confirms linearity, *r^2^* values ranging between .943 and .999. Thus, there seems to be no need to incorporate a third parameter in the model (with reference to Kornbrot, 2016). Furthermore, this finding comes in complete support of one goal of the present study, namely to verify that performance on our TP task can be aptly fitted by the psychophysical function.

**Figure 2:**
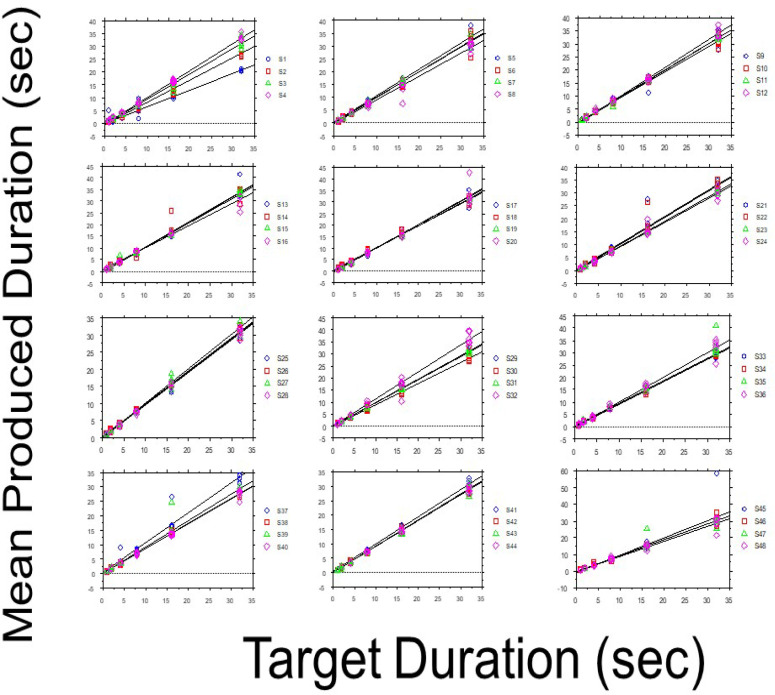
Mean produced duration as a function of target duration for each of 48 sessions.

Sequential effects entailed by making multiple series of time productions manifest as a lengthening effect (longer time productions), as proposed in the literature (Ross, 1969; Ryan, 2012; Vroon, 1972, 1976; Vroon & van Boxtel, 1972). Figure 2 presents mean *P* as a function of *T* (i.e., prior to log transformation of the values) for each of the 48 sessions (S). As seen in the upper left panel of the figure, there is a clear lengthening effect over the first 4 sessions.^3^

Subsequent to these, one sees much less variability over time (i.e., more overlap of the linear regressions). This finding suggests that TP exhibits stability across sessions (within-session stability is addressed below) but does indicate that the first few sessions might need to be carefully examined for sequential effects.

What is the best way to analyze the data from this *n* = 1 study, having a factorial design? The answer, as the first author has both suggested and implemented (Glicksohn, 2004, p. 265; Glicksohn et al., 2019), is to pool interactions in order to create an error term^4^ to test for the 3 main effects of Flicker, Modality and Counting Strategy.

### Analyzing mean log(*P*)

**Figure 3:**
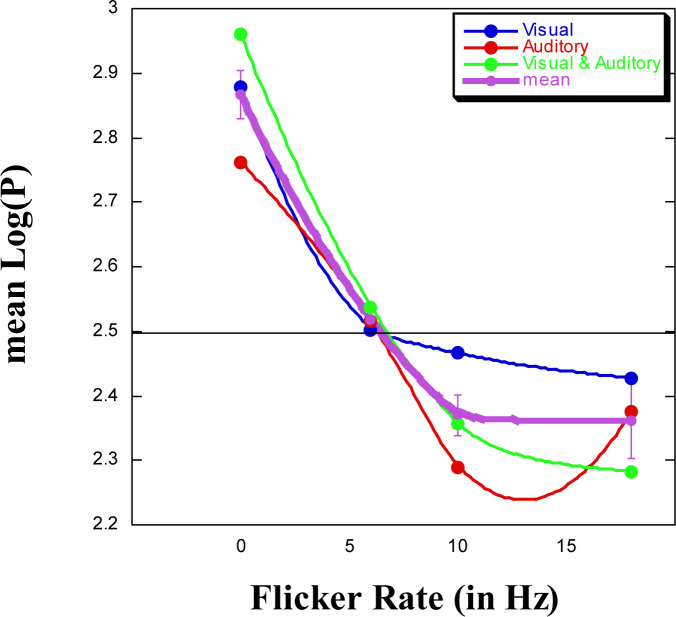
Mean log-transformed (to base 2) produced duration (± SE) as a function of flicker rate and of modality.

We ran a 4 (Flicker) x 3 (Modality) x 4 (Counting Strategy) analysis of variance (ANOVA) on mean log(*P*), pooling all the two-way interaction and the three-way interaction *SS* (sum of squares) values, and subsequently dividing this by their pooled *df* (degrees of freedom) to create a suitable mean square error term (*MSE*). The main effect for Flicker, having four conditions [*F*(3, 39) = 35.26, *MSE* = 0.019, *p* < .01] is clearly apparent (see Figure 3); there is no main effect for either Modality, having three conditions [*F*(2, 39) = 1.47, *ns*] or Counting Strategy, having four conditions [*F*(3, 39) < 1]. The data clearly show that with increase in flicker rate, mean log(*P*) decreases, indicating that as the internal clock speeds up (in line with the faster flicker rate), produced time is shortened. This finding indicates that TP will be reliably influenced by flicker rate. Given that the midpoint value for *T* is 2.5, we note that without flicker (flicker rate = 0 Hz), mean log(*P*) is the longest, at 6 Hz mean log(*P*) is at this midpoint value, and at 10 Hz already approaches its asymptotic value. Figure 3 also presents the data when partitioned by modality, suggesting that at 18 Hz there is an aberrant value for the condition of auditory stimulation.

### Analyzing the power function

**Figure 4:**
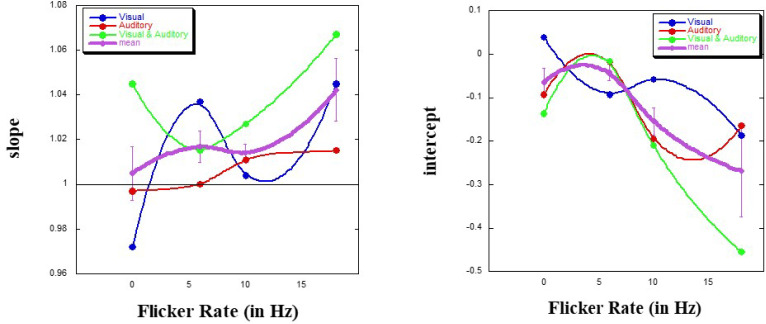
Slope and intercept of the psychophysical function (± SE) as a function of flicker rate and of modality.

We ran the same ANOVA on both the slope and the intercept of the within-session regressions of *P* on *T*, after log transformation of both. As before, we subsequently pooled all interactions to create suitable *MSE* values for each analysis. For the slope, it was a main effect for Modality [*F*(2, 39) = 5.00, *MSE* = 0.001, *p* < .05] which was uncovered (see Figure 4), with no effect for either Flicker [*F*(3, 39) = 2.73, *ns*] or Counting Strategy [*F*(2, 39) < 1]. The data show that the combined visual and auditory stimulation result in the highest slope. Further inspection of the data revealed that counting up at a flicker rate of 18 Hz when exposed to both visual and auditory stimulation produced aberrant data.^5^ In any event, given that the slope is practically 1.00 here, the locus of the effect of flicker is not in the slope, which reflects the exponent of the psychophysical power function. On the other hand, the data presented in Figure 4 suggest that there might well be an interaction between Flicker and Modality, worthy of consideration in future work.

There is no evidence that the size of the exponent (here, the slope) tends to increase with practice (Allan, 1983, p. 30). As opposed to the notion that when counting the slope would be close to 1, whereas when not counting this would be closer to 0.5 (Michon, 1985, p. 38), this is far from the case.

For the intercept, it was a main effect for Flicker [*F*(3, 39) = 3.32, *MSE* = 0.039, *p* < .05] which was uncovered (see Figure 4), with no effect for either Modality [*F*(2, 39) = 1.81, *ns*] or Counting Strategy [*F*(3, 39) < 1]. The data clearly show that with increase in flicker rate the intercept decreases, but that also the largest *SD* value is found at a flicker rate of 18 Hz. Again, this is due to one particular condition, as noted above. Furthermore, as for the slope, there might well be an interaction between Flicker and Modality, worthy of further investigation.

### Analyzing SD and CV

**Figure 5:**
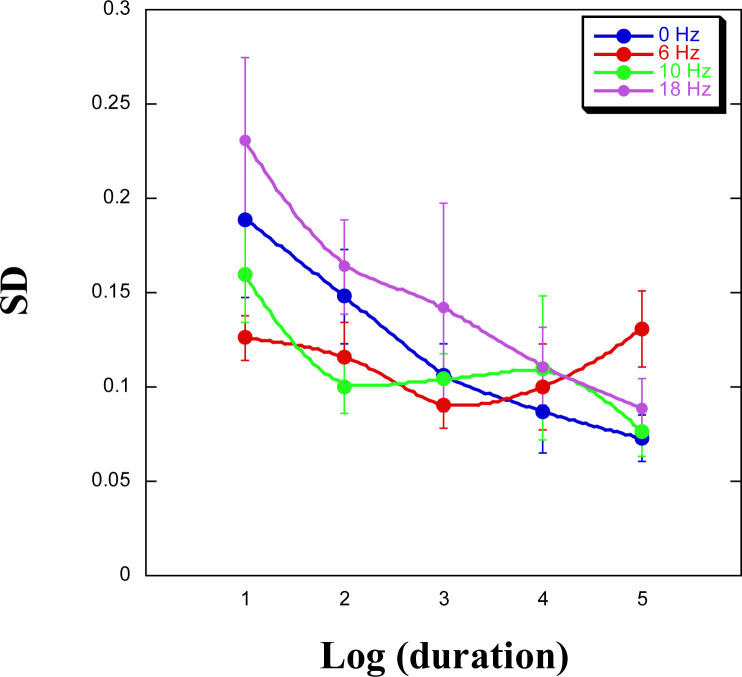
SD as a function of log-transformed (to base 2) target duration and flicker rate.

How does *SD* (of these log-transformed data) change as a function of target duration and flicker rate? Figure 5 presents these data. Clearly, *SD* decreases as a function of target duration, having five values (excluding that of one second) [*F*(4, 136) = 7.16, *MSE* = 0.007, *p* < .0001], with no significant effect of Flicker (neither main effect, nor interaction, both *F*-values < 1.4). Thus, as Allan (1983, p. 31) noted, “within-subject variance of the log responses is not homogeneous across stimulus values” and “… tends to decrease with response magnitude” (p. 35). When we looked at *SD* as a function of target duration and Counting Strategy, the main effect for target duration remained significant [*F*(4, 136) = 6.99, *MSE* = 0.007, *p* < .0001, with no significant effect of Counting Strategy (neither main effect, nor interaction, both *F*-values < 1.5].

Turning now to the coefficient of variation (CV), this measure decreases as a function of target duration [*F*(4, 136) = 6.0, *MSE* = 0.001, *p* = .0002, with no significant effect of Flicker (neither main effect, nor interaction, both *F*-values < 1.9].

### Between-session and within-session stability

**Figure 6:**
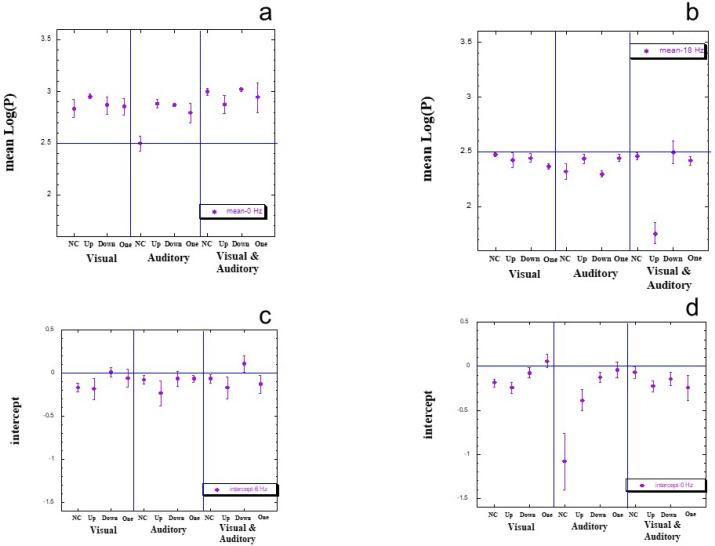
Between-session and within-session plots for mean log(P) and intercept values of TP. NC = not counting; Up = counting up to the target duration; Down = counting down to the target duration; One = counting ‘one’.

Our last analysis looks at both between-session and within-session stability of our TP data, taking into consideration the main effects reported above for each index. For mean log(*P*), it was only the main effect for flicker rate that was significant. Figure 6 presents between-session means for two select flicker rates, 0 Hz (panel a) and 18 Hz (panel b). At each flicker rate, one notes 12 separate series (appearing on the *X* axis), defined by crossing Modality and Counting Strategy. For each such series, we have plotted the mean value together with standard error (*SE*) based on the six separate series of the original 6 × 6 Latin Square of TP data for that session.

Consider Figure 6a, which indicates some degree of variability within each session, but also quite a degree of stability across sessions. The data point for the session of auditory flicker coupled with no counting (NC) clearly departs from this pattern of stability. Now compare Figure 6b to Figure 6a. Apart from the fact that all mean values are much lower (indicative of the main effect for flicker, reported above), note both the generally much higher stability within session, but also the aberrant data point for the session of visual & auditory flicker coupled with counting up. In general, however, we stress the high degree of both between- and within-session stability for mean log(P).

Figures 6c and 6d present the intercept means at two particular flicker frequencies of 6 and 0 Hz, respectively. Again, note the high degree of between-session stability for the intercept at 6 Hz flicker stimulation, and the somewhat varying degree of within-session stability. Note, further, that the intercept is usually negative (as already seen in Figure 2, above). For flicker at 0 Hz (Figure 6d) note that this is also quite stable, though there is an aberrant value for the session of auditory flicker coupled with not counting.

In general, then, both mean and intercept values exhibit a good degree of stability both within and between sessions. Indeed, it is because of such stability that aberrant data points can be easily seen, reflecting the source of experimental effects.

## Discussion

What are our conclusions from this study? We will present four. First, as clearly shown in Figure 2, when flicker rate increases, produced duration decreases, as predicted. We are intrigued by the fact that veridical perception is achieved at a flicker rate of 6 Hz. Allman et al. (2014, p.749) have recently suggested that in order “to measure the absolute speed of an internal clock”, one could “attempt to synchronize the time base with a repetitive signal (e.g., visual flicker or auditory click trains) presented at a known frequency.” Does the 6 Hz flicker fulfil this expectation? We recall that participants employing chronometric counting might well be instantiating an internal clock, in the sense that “the movement of the vocal apparatus, with its resonant frequency around 4 Hz, constitutes the pacemaker; the number system constitutes the register; the initiation of counting in response to the interval onset constitutes gating; the matching of the counts registered with a target constitutes the comparison” (Bizo, Chu, Sanabria, & Killeen, 2006, p. 201). We also note that between-subject differences in tapping rate ranged between 0.8 and 6 Hz in Fetterman and Killeen’s (1990, p. 769) study investigating such an internal clock model. We might also suggest that the value of 12.4 Hz, stressed by Treisman et al. (1990, p. 728) as perhaps constituting a “characteristic frequency” of the temporal oscillator component of the pacemaker, is itself twice the value of 6 Hz. We therefore suggest that this 6 Hz flicker rate would be worthy of further research attention.

Our second conclusion from this study relates to the psychophysical function underlying produced duration. The same main effect for flicker was found for the intercept, but not for the slope of the function. The slope estimate was practically 1.00, implicating an exponent of 1. Eisler (1996, p. 80) has suggested that “when durations are indicated by a series of regular intermittent stimuli (e.g. clicks), the exponent is close to one…. I interpret this finding as a constant resetting of the biological clock, counteracting the deceleration that takes place when a duration is indicated by a continuous stimulus.” Thus, the decrease in produced duration implicates a change in intercept value. We note that at a flicker rate of 6 Hz, the intercept approaches a value of 0 (hence, veridical perception; see Figure 4). That it is the intercept, and not the slope of this psychophysical function for TP that is influenced by flicker rate, is a finding supporting our previous reports that exposure to an altered sensory environment will impact specifically on the intercept (Glicksohn, 1992, 1996).

Various authors have highlighted the role that the study of flicker can have in advancing our knowledge of the pacemaker function of the internal clock (Herbst & Landau, 2016; Matell & Meck, 2004, p. 145). Whether one should assume a common (amodal) internal clock (Allan, 1979, p. 347; Ono, Horii, & Watanabe, 2012), or perhaps multiple clocks, one for each modality, each with a modality-specific pacemaker (Gorea, 2011; Yuasa & Yotsumoto, 2015) is still under debate (Klink et al., 2011; Levitan et al., 2015; Mayer et al., 2014). Our results indicate no modality effect for either mean log(*P*) or for the intercept, but only for the slope – and even then there is no marked deviation from the value of 1.00.

Nevertheless, looking closely at Figure 4, and pinpointing the 6 Hz flicker rate (as discussed above), one notes a high discrepancy between the slope values for visual (V) and for auditory (A) flicker, with V > A, and that for A being veridical (slope = 1). What might this reflect? Slightly longer *P* for visual flicker at 6 Hz, given a slightly larger slope for V, indicating a slower pacemaker rate for V, is somewhat supportive of the ‘auditory dominance’ assertion. But note further from Figure 4 that VA flicker results in a slope midway between that of V and that of A. This does not quite support the hypothesis of auditory dominance, that is, “when an auditory-visual compound is presented, pacemaker rate is determined by the auditory stimulus” (Wearden et al., 1998, p. 116). Nevertheless, one should recall that auditory dominance might only be seen “when both modalities are presented to the same participant in the same session” (Ortega et al., 2009, p. 270; see also Penney et al., 2000). Thus, our present design cannot help to resolve this issue.

What we can do is to reflect on the shape of the function appearing in Figure 3, noting the similarity of the visual and auditory functions. Yuasa and Yotsumoto (2015), assessing modality-specific pacemakers, argued that “the auditory pacemaker normally operates faster than 10.9 Hz, while the visual pacemaker normally operates slower than 10.9 Hz” (pp. 11-12). If this is so, then in our Figure 3 we should see at around 10 Hz that mean log(*P*) for V is longer than that for A – and this is exactly the case. But one further contribution of the present study is to suggest that the critical pacemaker rate differentiating between V and A probably lies between 6 and 10 Hz.

A second contribution that we can provide concerns the question recently raised by Herbst et al. (2013) regarding “whether the effect of flicker on perceived duration remains at a stable plateau or decreases at frequencies faster than 12 Hz.” From our Figure 3, it would seem that we can support the notion of a plateau—especially for visual flicker.

Returning now to the conclusions to be drawn from this study, our third conclusion relates to the impact of counting (or not) on time production. Much has been written about this in the literature (Allman et al., 2014; Rattat & Droit-Volet, 2012). Our modest suggestion here is that perhaps too much attention has been paid to the possible impact of chronometric counting on the (supposedly) natural functioning of the internal clock. In the present study, chronometric counting was entered as a planned factor in the design, and we employed no less than three different ways of counting. And yet we uncovered no main effect for counting. At the very least, we can argue that flicker swamps any impact of chronometric counting. Or, in line with Eisler’s (1996) comments quoted above, we can consider that chronometric counting might very well impact on the estimation of a duration indicated by a “continuous stimulus”, but not on one marked by flicker. We further note that at least in one study employing time production, performance during a ‘timing’ condition (the participants were requested to refrain from chronometric counting) and during a ‘counting’ condition was positively correlated at .68 (Bartholomew et al., 2015). Given this, it might well be irrelevant whether the positive correlation that we reported between hemispheric asymmetry in EEG peak alpha frequency and time production (Glicksohn et al., 2009) is “mediated” by chronometric counting – as recently argued by Venskus and Hughes (2021).

A fourth conclusion relates to the finding that the CV decreases as a function of target duration. True, the CV is computed on the log-transformed data. Nevertheless, given that CV decreases, as opposed to remaining constant, this is in strict opposition to SET employed with sub-second durations (Grondin, 2001, p. 29), but is in line with what is known about chronometric counting and longer time intervals (Clément & Droit-Volet, 2006, p. 165; Hancock & Rausch, 2010, p. 175; Wearden, 1991, p. 63).

We address now a number of limitations of the present study. First, the range of durations examined here has excluded those lasting less than one second. While this might be considered to be a limitation, one should also consider the notion that the perception of very brief durations will have little relevance for the perception of the longer durations employed here (Grondin, 2001; Matthews & Meck, 2016). Indeed, as Jiří Wackermann (2007, p. 29) has written, “Can we really speak about the experience of a duration so short that the participants do not even have time to utter ‘one, two’ in their minds?”. In line with this, even the inclusion of the onesecond target duration is questionable, especially given the fact that we have missing data for this. Furthermore, there should also be no particular benefit for counting for this target duration (Grondin et al., 1999). Future studies using the present experimental protocol might therefore consider fore-going this particular target duration. A second limitation could be the inclusion of the counting ‘one’ condition, in that repeating ‘one’ does not, strictly speaking, comprise chronometric counting – though it does constitute a neat control for the other counting conditions. A third limitation is that the effect of counting has not been addressed with respect to the recruitment of the phonological loop. While the theoretical connection between counting and the phonological loop has been noted in the literature (Fortin & Breton, 1995; Wiener, Turkeltaub, & Coslett, 2010), the main issue here is not in terms of phenomenology, nor electrophysiology, but rather whether chronometric counting is qualitatively (and quantitatively) different from timing with no counting (e.g., Glicksohn & Berkovich-Ohana, 2019). Given that chronometric counting did not appear to impact on our results, these two limitations can, however, be downplayed.

A fourth limitation to consider is that in each session, each particular target duration was produced only six times. Yet, as Diana Kornbrot (Kornbrot et al., 2013, pp. 7-8) has suggested “Good estimates of functional form require judgments of a minimum of 5 different physical values. It’s our view, that one gets power per pound … by increasing the number of time intervals to be estimated than by having several replications of each estimate (but we have not yet tested this mathematically)”. A fifth limitation to note is that there is no focus on entrainment to the flicker rate (Allman et al., 2014; Brighouse et al., 2014; Herbst et al., 2013; Matell & Meck, 2004; Teghil et al., 2019; Wearden et al., 2017; Wiener & Kanai, 2016), and this is certainly a topic to be addressed in future studies using this experimental protocol, using a reasonable sample size. Clearly, sample size is the major limitation of the present study, which, as a pilot *n* = 1 study, has achieved its objectives: showing the efficacy of the experimental protocol; demonstrating the quality of the data that can be generated. The study needs to be replicated; more data are needed; individual differences in performance – especially while exposed to flicker – due to personality (Glicksohn & Naftuliev, 2005) and/or cognitive style (Teghil et al., 2019) need to be considered. Clearly, there is much to explore here.

## Footnotes

1 It is important to stress our division of labor in preparing this manuscript. Both authors planned the study. The second author ran the study on herself and prepared the raw data for analysis. The first author analyzed the data and wrote the manuscript. Hence, data collection and data analysis have been handled separately, in order to reduce as far as feasibly possible the spectre of experimenter bias. Furthermore, the second author was a visiting student at the time the study was run, with no prior knowledge in the field. Indeed, she had practically no research experience at the time, and certainly not in this area of time perception. Hence, we can rule out the possibility that her own performance on the time production task was somehow influenced by any prior knowledge of the research literature, or that her produced data would be anything other than that produced by any other naïve participant.

2 Note that when a participant remains with eyes closed, flicker stimulation has a clear impact on brain activity. Indeed, using the same flicker stimulation, a driving response of alpha activity (8-13 Hz) can be clearly elicited at the 10 Hz flicker rate (Glicksohn 1986-87). With eyes closed, a participant in this type of study is better able to focus on the task at hand.

3 These first 4 sessions (S) were as follows: S1 = visual (V) and auditory (A) 18 Hz flicker, coupled with counting up; S2 = VA 10 Hz flicker, coupled with counting up; S3 = VA 18 Hz flicker, coupled with counting “one”‘ and S4 = VA 6 Hz flicker, coupled with counting down. We will subsequently check whether our results are overtly influenced by S1 and S3.

4 See also von Eye and Wiedermann (2015), for a full discussion of this and other issues concerning the analysis of such data.

5 This aberrant data point comes from S1. The slope for this session was 1.18.

## Action editor

Lars-Gunnar Lundh served as action editor for this article.

## References

[cit0001] Allan, L. G. (1979). The perception of time. *Perception & Psychophysics*, 26(5), 340–354. 10.3758/BF03204158

[cit0002] Allan, L. G. (1983). Magnitude estimation of temporal intervals. *Perception & Psychophysics*, 33(1), 29–42. 10.3758/BF03205863 6844090

[cit0003] Allman, M. J., Teki, S., Griffiths, T. D., & Meck, W. H. (2014). Properties of the internal clock: First- and second-order principles of subjective time. *Annual Review of Psychology*, 65, 743–771. 10.1146/annurev-psych-010213-115117 24050187

[cit0004] Baldauf, D., Burgard, E., & Wittmann, M. (2009). Time perception as a workload measure in simulated car driving. *Applied Ergonomics*, 40(5), 929–935. 10.1016/j.apergo.2009.01.004 19200943

[cit0005] Bartholomew, A. J., Meck, W. H., & Cirulli, E. T. (2015). Analysis of genetic and non-genetic factors influencing timing and time perception. *PLoS ONE*, 10, e0143873. 10.1371/journal.pone.0143873 26641268 PMC4671567

[cit0006] Baudouin, A., Vanneste, S., Isingrini, M., & Pouthas, V. (2006). Differential involvement of internal clock and working memory in the production and reproduction of duration: A study on older adults. *Acta Psychologica*, 121(3), 285–296. 10.1016/j.actpsy.2005.07.004 16139783

[cit0007] Binkofski, F., & Block, R. A. (1996). Accelerated time experience after left frontal cortex lesion. *Neurocase*, 2(6), 485–493. 10.1080/13554799608402424

[cit0008] Bizo, L. A., Chu, J. Y. M., Sanabria, F., & Killeen, P. R. (2006). The failure of Weber’s law in time perception and production. *Behavioural Processes*, 71(2-3), 201–210. 10.1016/j.beproc.2005.11.006 16386378

[cit0009] Boltz, M. G. (1994). Changes in internal tempo and effects on the learning and remembering of event durations. *Journal of Experimental Psychology: Learning, Memory, and Cognition*, 20(5), 1154–1171. 10.1037/0278-7393.20.5.1154 1402718

[cit0010] Brighouse, C., Hartcher-O’Brien, J., & Levitan, C. A. (2014). Encoding of duration and rate by an integrative model of temporal processing. *Timing & Time Perception Reviews*, 1, article 3. 10.1163/24054496-00101003

[cit0011] Buhusi, C. V., & Meck, W. H. (2009). Relativity theory and time perception: single or multiple clocks? *PLoS ONE*, 4, e6268. 10.1371/journal.pone.0006268 19623247 PMC2707607

[cit0012] Chen, K.-M., & Yeh, S.-L. (2009). Asymmetric cross-modal effects in time perception. *Acta Psychologica*, 130, 225–234. 10.1016/j.actpsy.2008.12.008 19195633

[cit0013] Clément, A., & Droit-Volet, S. (2006). Counting in a time discrimination task in children and adults. *Behavioural Processes*, 71(2-3), 164–171. 10.1016/j.beproc.2005.08.007 16434150

[cit0014] Coelho, M., Ferreira, J. J., Dias, B., Sampaio, C., Martins, I. P., & Castro-Caldas, A. (2004). Assessment of time perception: The effect of aging. *Journal of the International Neuropsychological Society*, 10(3), 332–341. 10.1017/S1355617704103019 15147591

[cit0015] Droit-Volet, S. (2014). What emotions tell us about time. In D. Lloyd & V. Arstila (Eds.), *Subjective time: The philosophy, psychology, and neuroscience of temporality* (pp. 477–506). Cambridge, MA: MIT Press.

[cit0016] Eisler, H. (1976). Experiments on subjective duration 1868-1975: A collection of power function exponents. *Psychological Bulletin*, 83(6), 1154–1171. 10.1037/0033-2909.83.6.1154 996212

[cit0017] Eisler, H. (1996). Time perception from a psychophysicist’s perspective. In H. Helfrich (Ed.), *Time and mind* (pp. 65–86). Seattle: Hogrefe & Huber.

[cit0018] Fetterman, J. G., & Killeen, P. R. (1990). A componential analysis of pacemaker-counter timing systems. *Journal of Experimental Psychology: Human Perception and Performance*, 16(4), 766–780. 10.1037/0096-1523.16.4.766 2148591

[cit0019] Fortin, C., & Breton, R. (1995). Temporal interval production and processing in working memory. *Perception & Psychophysics*, 57(2), 203–215. 10.3758/BF03206507 7885819

[cit0020] Gibbon, J., Malapani, C., Dale, C. L., & Gallistel, C. R. (1997). Toward a neurobiology of temporal cognition: Advances and challenges. *Current Opinion in Neurobiology*, 7(2), 170–184. 10.1016/S0959-4388(97)80005-0 9142762

[cit0021] Glicksohn, J. (1986-87). Photic driving and altered states of consciousness: An exploratory study. *Imagination, Cognition and Personality*, 6(2), 167–182. 10.2190/5K21-DDWL-E66B-2CXE

[cit0022] Glicksohn, J. (1992). Subjective time estimation in altered sensory environments. *Environment and Behavior*, 24(5), 634–652. 10.1177/0013916592245004

[cit0023] Glicksohn, J. (1996). Entering trait and context into a cognitive-timer model for time estimation. *Journal of Environmental Psychology*, 16(4), 361–370. 10.1006/jevp.1996.0030

[cit0024] Glicksohn, J. (2001). Temporal cognition and the phenomenology of time: A multiplicative function for apparent duration. *Consciousness and Cognition*, 10(1), 1–25. 10.1006/ccog.2000.0468 11273623

[cit0025] Glicksohn, J. (2004). From methodology to data analysis: Prospects for the *n* = 1 intrasubject design. *Behavioral and Brain Sciences*, 27(2), 264–266. 10.1017/S0140525X04250063 18241493

[cit0026] Glicksohn, J., & Berkovich-Ohana, A. (2019). When meditators avoid counting during time production things get interesting. *PsyCh Journal*, 8(1), 17–27. 10.1002/pchj.250 30358176

[cit0027] Glicksohn, J., Berkovich-Ohana, A., Balaban Dotan, T., Goldstein, A., & Donchin, O. (2009). Time production and EEG alpha revisited. *NeuroQuantology*, 7(1), 138–151. 10.14704/nq.2009.7.1.215

[cit0028] Glicksohn, J., Berkovich-Ohana, A., Mauro, F., & Ben-Soussan, T. D. (2019). Individual EEG alpha profiles are gender-dependent and indicate subjective experiences in whole-body perceptual deprivation. *Neuropsychologia*, 125, 81–92. 10.1016/j.neuropsychologia.2019.01.018 30711610

[cit0029] Glicksohn, J., & Hadad, Y. (2012). Sex differences in time production revisited. *Journal of Individual Differences*, 33(1), 35–42. 10.1027/1614-0001/a000059

[cit0030] Glicksohn, J., & Naftuliev, Y. (2005). In search of an electrophysiological index for psychoticism. *Personality and Individual Differences*, 39(6), 1083–1092. 10.1016/j.paid.2005.07.013

[cit0031] Gorea, A. (2011). Ticks per thought or thoughts per tick? A selective review of time perception with hints on future research. *Journal of Physiology-Paris*, 105, 153–163. 10.1016/j.jphysparis.2011.09.008 21963529

[cit0032] Grondin, S. (2001). From physical time to the first and second moments of psychological time. *Psychological Bulletin*, 127(1), 22–44. 10.1037/0033-2909.1127.1031.1022 11271754

[cit0033] Grondin, S., Meilleur-Wells, G., & Lachance, R. (1999). When to start explicit counting in a time-intervals discrimination task: A critical point in the timing process of humans. *Journal of Experimental Psychology: Human Perception and Performance*, 25(4), 993–1004. 10.1037/0096-1523.25.4.993

[cit0034] Grondin, S., Ouellet, B., & Roussel, M.-E. (2004). Benefits and limits of explicit counting for discriminating temporal intervals. *Canadian Journal of Experimental Psychology*, 58(1), 1–12. 10.1037/h0087436 15072205

[cit0035] Hancock, P. A., & Block, R. A. (2016). A new law for time perception. *American Journal of Psychology*, 129(2), 111–124. 10.5406/amerjpsyc.129.2.0111 27424414

[cit0036] Hancock, P. A., & Rausch, R. (2010). The effects of sex, age, and interval duration on the perception of time. *Acta Psychologica*, 133(2), 170–179. 10.1016/j.actpsy.2009.11.005 20018272

[cit0037] Herbst, S. K., Javadi, A. H., van der Meer, E., & Busch, N. A. (2013). How long depends on how fast—perceived flicker dilates subjective duration. *PLoS ONE*, 8(10), e76074. 10.1371/journal.pone.0076074 24194829 PMC3806760

[cit0038] Herbst, S. K., & Landau, A. N. (2016). Rhythms for cognition: the case of temporal processing. *Current Opinion in Behavioral Sciences*, 8, 85–93. 10.1016/j.cobeha.2016.01.014

[cit0039] Hicks, R. E., & Allen, D. A. (1979). The repetition effect in judgments of temporal duration across minutes, days, and months. *American Journal of Psychology*, 92(2), 323–333. 10.2307/1421927 474836

[cit0040] Hinton, S. C., Harrington, D. L., Binder, J. R., Durgerian, S., & Rao, S. M. (2004). Neural systems supporting timing and chronometric counting: An fMRI study. *Cognitive Brain Research*, 21(2), 183–192. 10.1016/j.cogbrainres.2004.04.009 15464350

[cit0041] Hinton, S. C., & Rao, S. M. (2004). “One-thousand one… one-thousand two… ”: Chronometric counting violates the scalar property in interval timing. *Psychonomic Bulletin & Review*, 11(1), 24–30. 10.3758/BF03206456 15116982

[cit0042] Kladopoulos, C. N., Hemmes, N. S., & Brown, B. L. (2004). Prospective timing under dual-task paradigms: Attentional and contextual-change mechanisms. *Behavioural Processes*, 67, 221–233. 10.1016/j.beproc.2003.12.004 15497256

[cit0043] Klink, P. C., Montijn, J. S., & van Wezel, R. J. (2011). Cross-modal duration perception involves perceptual grouping, temporal ventriloquism, and variable internal clock rates. *Attention, Perception, & Psychophysics*, 73(1), 219–236. 10.3758/s13414-010-0010-9 PMC3025116 21258921

[cit0044] Kornbrot, D. E. (2016). Human psychophysical functions, an update: Methods for identifying their form; estimating their parameters; and evaluating the effects of important predictors. *Psychometrika*, 81(1), 201–216. 10.1007/s11336-014-9418-9 25187323

[cit0045] Kornbrot, D. E., Msetfi, R. M., & Grimwood, M. J. (2013). Time perception and depressive realism: Judgment type, psychophysical functions and bias. *PLoS ONE*, 8(8), e71585. 10.1371/journal.pone.0071585 23990960 PMC3749223

[cit0046] Levitan, C. A., Ban, Y.-H. A., Stiles, N. R., & Shimojo, S. (2015). Rate perception adapts across the senses: evidence for a unified timing mechanism. *Scientific Reports*, 5(1), 8857. 10.1038/srep08857 25748443 PMC4894401

[cit0047] Matell, M. S., & Meck, W. H. (2004). Cortico-striatal circuits and interval timing: Coincidence detection of oscillatory processes. *Cognitive Brain Research*, 21(2), 139–170. 10.1016/j.cogbrainres.2004.06.012 15464348

[cit0048] Matthews, W. J., & Meck, W. H. (2016). Temporal cognition: Connecting subjective time to perception, attention, and memory. *Psychological Bulletin*, 142(8), 865–907. 10.1037/bul0000045 27196725

[cit0049] Mayer, K. M., Di Luca, M., & Ernst, M. O. (2014). Duration perception in crossmodally-defined intervals. *Acta Psychologica*, 147, 2–9. 10.1016/j.actpsy.2013.07.009 23953664

[cit0050] McAuley, J. D., & Jones, M. R. (2003). Modeling effects of rhythmic context on perceived duration: A comparison of interval and entrainment approaches to short-interval timing. *Journal of Experimental Psychology: Human Perception and Performance*, 29, 1102–1125. 10.1037/0096-1523.29.6.1102 14640833

[cit0051] Meck, W. H., & Benson, A. M. (2002). Dissecting the brain’s internal clock: how frontal–striatal circuitry keeps time and shifts attention. *Brain and Cognition*, 48(1), 195–211. 10.1006/brcg.2001.1313 11812042

[cit0052] Michon, J. A. (1985). The compleat time experiencer. In J. A. Michon & J. L. Jackson (Eds.), *Time, mind, and behavior* (pp. 20–52). New York: Springer.

[cit0053] Mimura, M., Kinsbourne, M., & O’Connor, M. (2000). Time estimation by patients with frontal lesions and by Korsakoff amnesics. *Journal of the International Neuropsychological Society*, 6(5), 517–528. 10.1017/S1355617700655017 10932471

[cit0054] Miró, E., Cano, M. C., Espinosa-Fernández, L., & Buela-Casal, G. (2003). Time estimation during prolonged sleep deprivation and its relation to activation measures. *Human Factors*, 45(1), 148–159. 10.1518/hfes.45.1.148.27227 12916587

[cit0055] Myers, P. M., & Tilley, A. (2003). The relationship between diurnal type and time duration estimation at morning and evening times of day. *Personality and Individual Differences*, 35(5), 1141–1150. 10.1016/S0191-8869(02)00324-0

[cit0056] Ono, F., Horii, S., & Watanabe, K. (2012). Individual differences in vulnerability to subjective time distortion. *Japanese Psychological Research*, 54(2), 195–201. 10.1111/j.1468-5884.2011.00497.x

[cit0057] Ortega, L., Lopez, F., & Church, R. M. (2009). Modality and intermittency effects on time estimation. *Behavioural Processes*, 81(2), 270–273. 10.1016/j.beproc.2009.02.009 19429221

[cit0058] Penney, T. B., Gibbon, J., & Meck, W. H. (2000). Differential effects of auditory and visual signals on clock speed and temporal memory. *Journal of Experimental Psychology: Human Perception and Performance*, 26(6), 1770–1787. 10.1037/0096-1523.26.6.1770 11129373

[cit0059] Penton-Voak, I. S., Edwards, H., Percival, A., & Wearden, J. H. (1996). Speeding up an internal clock in humans? Effects of click trains on subjective duration. *Journal of Experimental Psychology: Animal Behavior Processes*, 22(3), 307–320. 10.1037/0097-7403.22.3.307 8691161

[cit0060] Rattat, A.-C., & Droit-Volet, S. (2012). What is the best and easiest method of preventing counting in different temporal tasks? *Behavior Research Methods*, 44(1), 67–80. 10.3758/s13428-011-0135-3 21789731

[cit0061] Ross, D. A. (1969). Lengthening of time estimates in four different temporal patterns of visual light stimulation. *Psychonomic Science*, 16(4), 194–195. 10.3758/BF03336373

[cit0062] Rule, S. J. (1993). Analyzing coefficients of psychophysical power functions. *Perception & Psychophysics*, 54(4), 439–445. 10.3758/BF03211766 8255706

[cit0063] Ryan, L. J. (2012). Context effects and selective response lengthening in a temporal serial reproduction task. *Journal of Cognitive Psychology*, 24(5), 497–510. 10.1080/20445911.2012.658038

[cit0064] Ryan, L. J., Henry, K., Robey, T., & Edwards, J. A. (2004). Resolution of conflicts between internal and external information sources on a time reproduction task: The role of perceived information reliability and attributional style. *Acta Psychologica*, 117(2), 205–229. 10.1016/j.actpsy.2004.06.005 15464014

[cit0065] Teghil, A., Boccia, M., & Guariglia, C. (2019). Field dependence–independence differently affects retrospective time estimation and flicker-induced time dilation. *Experimental Brain Research*, 237, 1019–1029. 10.1007/s00221-019-05485-3 30729268

[cit0066] Treisman, M. (1963). Temporal discrimination and the indifference interval: Implications for a model of the “internal clock”. *Psychological Monographs: General and Applied*, 77(13), Whole No. 576. 10.1037/h0093864 5877542

[cit0067] Treisman, M., & Brogan, D. (1992). Time perception and the internal clock: Effects of visual flicker on the temporal oscillator. *European Journal of Cognitive Psychology*, 4(1), 41–70. 10.1080/09541449208406242

[cit0068] Treisman, M., Cook, N., Naish, P. L. N., & MacCrone, J. K. (1994). The internal clock: Electroencephalographic evidence for oscillatory processes underlying time perception. *The Quarterly Journal of Experimental Psychology*, 47A(2), 241–289. 10.1080/14640749408401112 8036266

[cit0069] Treisman, M., Faulkner, A., Naish, P. L. N., & Brogan, D. (1990). The internal clock: Evidence for a temporal oscillator underlying time perception with some estimates of its characteristic frequency. *Perception*, 19(6), 705–743. 10.1068/p190705 2130371

[cit0070] van Rijn, H., & Taatgen, N. A. (2008). Timing of multiple over-lapping intervals: How many clocks do we have? *Acta Psychologica*, 129(3), 365–375. 10.1016/j.actpsy.2008.09.002 18849020

[cit0071] Venskus, A., & Hughes, G. (2021). Individual differences in alpha frequency are associated with the time window of multisensory integration, but not time perception. *Neuropsychologia*. 10.1016/j.neuropsychologia.2021.107919 34153304

[cit0072] von Eye, A., & Wiedermann, W. (2015). General linear models for the analysis of single subject data and for the comparison of individuals. *Journal for Person-Oriented Research*, 1(1-2), 56–71. 10.17505/jpor.2015.07

[cit0073] Vroon, P. A. (1972). The lengthening effect in sequential estimations of a short interval: An alternative explanation. *Psychologische Forschung*, 35(4), 263–276. 10.1007/BF00424550 4649605

[cit0074] Vroon, P. A. (1976). Sequential estimations of time. *Acta Psychologica*, 40(6), 475–487. 10.1016/0001-6918(76)90025-1 998327

[cit0075] Vroon, P. A., & van Boxtel, A. (1972). Testing some implications of the sensory physiological model of the time sense. *Psychologische Forschung*, 35(2), 81–92. 10.1007/BF00416485 5054999

[cit0076] Wackermann, J. (2007). Inner and outer horizons of time experience. *The Spanish Journal of Psychology*, 10(1), 20–32. 10.1017/S1138741600006284 17549875

[cit0077] Wearden, J. H. (1991). Do humans possess an internal clock with scalar timing properties? *Learning and Motivation*, 22(1-2), 59–83. 10.1016/0023-9690(1091)90017-90013

[cit0078] Wearden, J. (2016). *The psychology of time perception*. London: Palgrave Macmillan.

[cit0079] Wearden, J. H., & Culpin, V. (1998). Exploring scalar timing theory with human subjects. In V. De Keysar, G. d’Ydewalle, & A. Vandierendonck (Eds.), *Time and the dynamic control of behavior* (pp. 33–49). Seattle: Hogrefe & Huber.

[cit0080] Wearden, J. H., Edwards, H., Fakhri, M., & Percival, A. (1998). Why “sounds are judged longer than lights”: Application of a model of the internal clock in humans. *The Quarterly Journal of Experimental Psychology*, 51B(2), 97–120. 10.1080/713932672 9621837

[cit0081] Wearden, J. H., Williams, E. A., & Jones, L. A. (2017). What speeds up the internal clock? Effects of clicks and flicker on duration judgements and reaction time. *The Quarterly Journal of Experimental Psychology*, 70(3), 488–503. 10.1080/17470218.2015.1135971 26811017

[cit0082] Wiener, M., & Kanai, R. (2016). Frequency tuning for temporal perception and prediction. *Current Opinion in Behavioral Sciences*, 8, 1–6. 10.1016/j.cobeha.2016.01.001

[cit0083] Wiener, M., Turkeltaub, P., & Coslett, H. B. (2010). The image of time: A voxel-wise meta-analysis. *NeuroImage*, 49(2), 1728–1740. 10.1016/j.neuroimage.2009.09.064 19800975

[cit0084] Yuasa, K., & Yotsumoto, Y. (2015). Opposite distortions in interval timing perception for visual and auditory stimuli with temporal modulations. *PLoS ONE*, 10(8), e0135646. https://doi.org/0.1371/journal.pone.0135646 26292285 10.1371/journal.pone.0135646 PMC4546296

[cit0085] Zakay, D. (1993). Time estimation methods—do they influence prospective duration estimates? *Perception*, 22(1), 91–101. 10.1068/p220091 8474838

[cit0086] Zakay, D., & Block, R. A. (1997). Temporal cognition. *Current Directions in Psychological Science*, 6(1), 12–16. 10.1111/1467-8721.ep11512604.

